# Multiple Comparison Analysis of Two New Genomic Sequences of ILTV Strains from China with Other Strains from Different Geographic Regions

**DOI:** 10.1371/journal.pone.0132747

**Published:** 2015-07-17

**Authors:** Yan Zhao, Congcong Kong, Yunfeng Wang

**Affiliations:** 1 Division of Avian Respiratory Disease Group, Harbin Veterinary Research Institute, The Chinese Academy of Agricultural Sciences, 427 Maduan Street, Harbin, China; 2 National Engineering Research Center of Veterinary Biologics, Harbin, China; The University of Melbourne, AUSTRALIA

## Abstract

To date, twenty complete genome sequences of ILTV strains have been published in GenBank, including one strain from China, and nineteen strains from Australian and the United States. To investigate the genomic information on ILTVs from different geographic regions, two additional individual complete genome sequences of WG and K317 strains from China were determined. The genomes of WG and K317 strains were 153,505 and 153,639 bp in length, respectively. Alignments performed on the amino acid sequences of the twelve glycoproteins showed that 13 out of 116 mutational sites were present only among the Chinese strain WG and the Australian strains SA2 and A20. The phylogenetic tree analysis suggested that the WG strain established close relationships with the Australian strain SA2. The recombination events were detected and confirmed in different subregions of the WG strain with the sequences of SA2 and K317 strains as parental. In this study, two new complete genome sequences of Chinese ILTV strains were used in comparative analysis with other complete genome sequences of ILTV strains from China, the United States, and Australia. The analysis of genome comparison, phylogenetic trees, and recombination events showed close relationships among the Chinese strain WG and the Australian strains SA2. The information of the two new complete genome sequences from China will help to facilitate the analysis of phylogenetic relationships and the molecular differences among ILTV strains from different geographic regions.

## Introduction

Infectious laryngotracheitis virus (ILTV) or Gallid herpesvirus 1 is a member of the family Herpesviridae according to the Ninth International Committee on the Taxonomy of Viruses [1]. ILTV can cause an acute, highly contagious upper-respiratory infectious disease in chickens [2, 3], resulting in economic losses in poultry industries worldwide [3, 4]. Even though much effort has been made in controlling ILT worldwide, outbreaks of this disease still occur. Partly because current vaccines may fail to protect against virulent field isolates. Another great concern is that virulent field viruses can be formed by natural recombination due to the use of attenuated vaccines [5–9]. It has been hypothesised that viral evolution in a geographically isolated environment could explain the high divergence between the Australian and European origin strains of ILTV [10]. Since the use of many ILTV vaccines are not limited to a particular country or region, the level of divergence between clinical isolates of ILTV from different locations is an interesting area of investigation. This requires a large number of complete genome sequence information of ILTV strains isolated from different period of time, different countries and even in different regions to perform a comparative analysis.

The ILTV genome was initially characterized by restriction endonuclease analysis [11–13]. Until 2006, the first complete genomic sequence of ILTV was assembled from overlapping genomic contigs of different strains [14–34]. So far, twenty complete genome sequences of ILTV strains have been determined using sanger sequencing techniques [35] and high-throughput sequencing techniques [10, 36–40]. The data of these complete sequences is available from GenBank. One strain is from China [35], twelve strains are from USA [9, 10, 36, 39, 40], whereas seven other strains are from Australia [37, 38]. Current available analyses are focused on the differences among live attenuated vaccine strains within Australia and the United States, respectively [10, 37], the differences among virulent strains isolates in the United States [38], and characteristics of vaccine strains and virulent strains in the two countries [36]. Therefore, acquisition of additional individual complete genome sequence information of ILTV strains from other countries will broaden our horizons.

This study aimed to analyze the evolutionary relationships, the molecular differences, and the recombination events among ILTV strains from different regions. In addition to the ILTV LJS09 strain sequences that we have submitted to GenBank, another two complete genome sequences of Chinese ILTV strains (WG, a virulent field strain, and K317, a vaccine strain) were obtained by conventional PCR and Sanger sequencing methods in this study. Comparative sequence analyses were performed using genome sequences of all the twenty-two ILTV strains.

## Materials and Methods

### Viruses

ILTV WG strain was a virulent strain isolated from WangGang of China in the 1950s, the WG virus used in this study was sixteenth passages from isolation, which was kindly provided by Harbin Weike Biotechnology Development Company (China). ILTV K317 strain was isolated from the commercialized live attenuated vaccine in China (Qingdao Yebio Bioengineering Company Limited, China). Viruses were propagated in embryonated eggs as reported previously[7].

### Cloning, sequencing and genome assembly

Viral DNA preparation and cloning of the viral genomes of ILTV WG and K317 strains were performed as described previously [35]. In brief, the majority of the sequences in the unique long region (U_L_) and unique short region (Us) of the viral genome were amplified by conventional PCR. The sequence in the terminal repeat regions (TRs)/U_L_ junction region was acquired by modified single oligonucleotide nested PCR (SON-PCR) [41]. The determination of the sequence in internal repeat regions (IRs) and TRs region was amplified using four pairs of specific primers, which matched the genome with single binding site to ensure the specificity and the directionality of the products. All the primers and PCR procedures used in this study were the same as previously reported [35].

The PCR products were cloned into pMD18-T vectors (TaKaRa, Dalian, China) and transformed into *E*. *coli* DH5a competent cells for selecting positive clones, which were sequenced by Shanghai Invitrogen Biotechnology Co. Ltd (Shanghai, China). To ensure the accuracy of the sequences, three positive clones of each fragment were selected for sequencing three times. DNA sequences were assembled using the Seqman program (DNASTAR, Madison, WI) and mapped manually. Open reading frames (ORFs) were predicted by the NCBI ORF Finder program and GeneMark program [42]. The complete sequences of the two strains were submitted to GenBank.

### Alignment and analysis

Twenty-two complete genome sequences of ILTV were used for comparison and analysis, of which twenty were obtained from GenBank, together with the two strains (WG and K317) newly obtained in this study. The information of twenty-two complete genome sequences are listed in Table 1. Alignments of complete genome sequences and that of the sequences in three different subregions (U_L_, Us, and IR, the TR was omitted as this region is the reverse complement of the IR) of ILTV strains were performed using the online program Multiple Alignment with Fast Fourier Transformation (MAFFT) version 7.0 software [http://mafft.cbrc.jp/alignment/software/] [43]. While partial genome sequence and the amino acid sequence alignments were performed using DNAMAN, Geneious software package and ClustalW program in MEGA5.1 [44]. Phylogenetic analysis (Maximum Parsimony method) on alignments of the sequences were conducted by the MEGA5.1 [44]. All phylogenetic reconstructions were assessed statistically by analyzing one hundred bootstrap replications.

**Table 1:** Comparison of the lengths of genomic subregions of the twenty-two ILTV strains.

Strain	Country/Pathotype	Accession No.	Total length
WG	China/virulent	JX458823	153505
K317	China/vaccine	JX458824	153639
LJS09	China/virulent	JX458822	153201
1874C5	USA/virulent	JN542533	149682
USDA	USA/virulent	JN542534	151756
81658	USA/virulent	JN542535	150335
63140/C/08/BR	USA/virulent	JN542536	153633
LT Blen	USA/vaccine ^a^	JQ083493	153623
Laryngo	USA/ vaccine ^a^	JQ083494	153624
CEO low passage	USA/CEO vaccine	JN580317	153641
CEO high passage	USA/CEO vaccine	JN580316	153647
CEO TRVX	USA/CEO vaccine	JN580313	153647
TCO low passage	USA/TCO vaccine	JN580315	155465
TCO high passage	USA/TCO vaccine	JN580314	150335
TCO IVAX	USA/TCO vaccine	JN580312	155465
SA2	Australia/vaccine ^a^	JN596962	152975
A20	Australia/vaccine ^a^	JN596963	152978
Serva	Australia/vaccine ^a^	HQ630064	152630
ACC78	Australia/virulent	JN804826	152632
CL-9	Australia/virulent	JN804827	152635
V1-99	Australia/virulent	JX646898	153630
CSW-1	Australia/virulent	JX646899	151671

^a^: vaccine = live attenuated vaccine

Recombination networks on alignments of the whole genome, and the sequences in different subregions (U_L_, Us, and IRs) of the twenty-two ILTV strains were performed by using SplitsTree 4 [45]. Statistical analysis of the recombination networks were generated by using the Phi test [46]. To further analyze the possibility of recombination, bootscan analysis was generated to detect the crossover points for recombination events of selected sequences as representatives of different cluster (WG, K317, LJS09, LT, USDA, 1874C5, Serva, CL-9, V1-99, and SA2 strains) using Simplot [47]. The informative site analysis and breakpoint analysis of the sequences in different subregions (U_L_, Us, and IRs) were performed using Simplot [47], with WG strain as query sequence, K317, SA2 as potential parental sequences and V1-99 strains as control sequence, respectively. The breakpoints analyses were further determined by DNAMAN software package.

## Results

### Sequencing and genomic organization

The genomes of the two Chinese ILTV strains sequenced by Sanger sequencing methods were assembled as 153505 bp (WG strain) and 153639 bp (K317 strain) in length, respectively. The U_L_, Us, and IRs (TRs were equal to IRs) of the two strains were 113155, 13613 and 13124 bp in length for strain WG, and 112913, 13816 and 13094 bp in length for strain K317 (Table 1), respectively. The two genome sequences have been deposited in GenBank [GenBank: JX458823 (WG); GenBank: JX458824 (K317)].

### Genome comparison in different subregions of the ILTV strains

**Fig 1:**
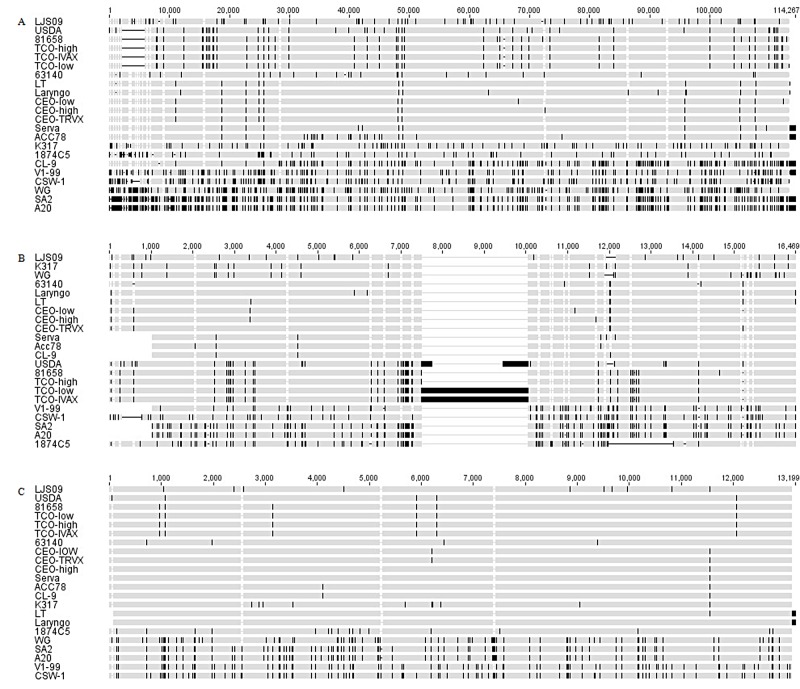
Genome comparison in different subregions of the ILTV strains. Comparison in U_L_ region (a), IRs region (b), and Us region (c) of all the twenty-two ILTV strains were performed by online program MAFFT and Geneious software package. Dashes indicate sequence deletions, and vertical lines indicate sequences with single nucleotide polymorphism (SNPs).

Comparison of the U_L_ regions of all the twenty-two ILTV strains showed identity of 98.6%. Sixteen of the twenty-two ILTV strains were about 113–114 kb in length. Another five strains from the United States (USDA, 81658, and the three TCO strains) contained a deletion of 3584 bp in the 5’ non-coding region of the genome and were 109 kb in length. While the CSW-1 strain from Australia was approximately 111 kb with a 1227 bp deletion in the same area (Fig 1A).

**Fig 2:**
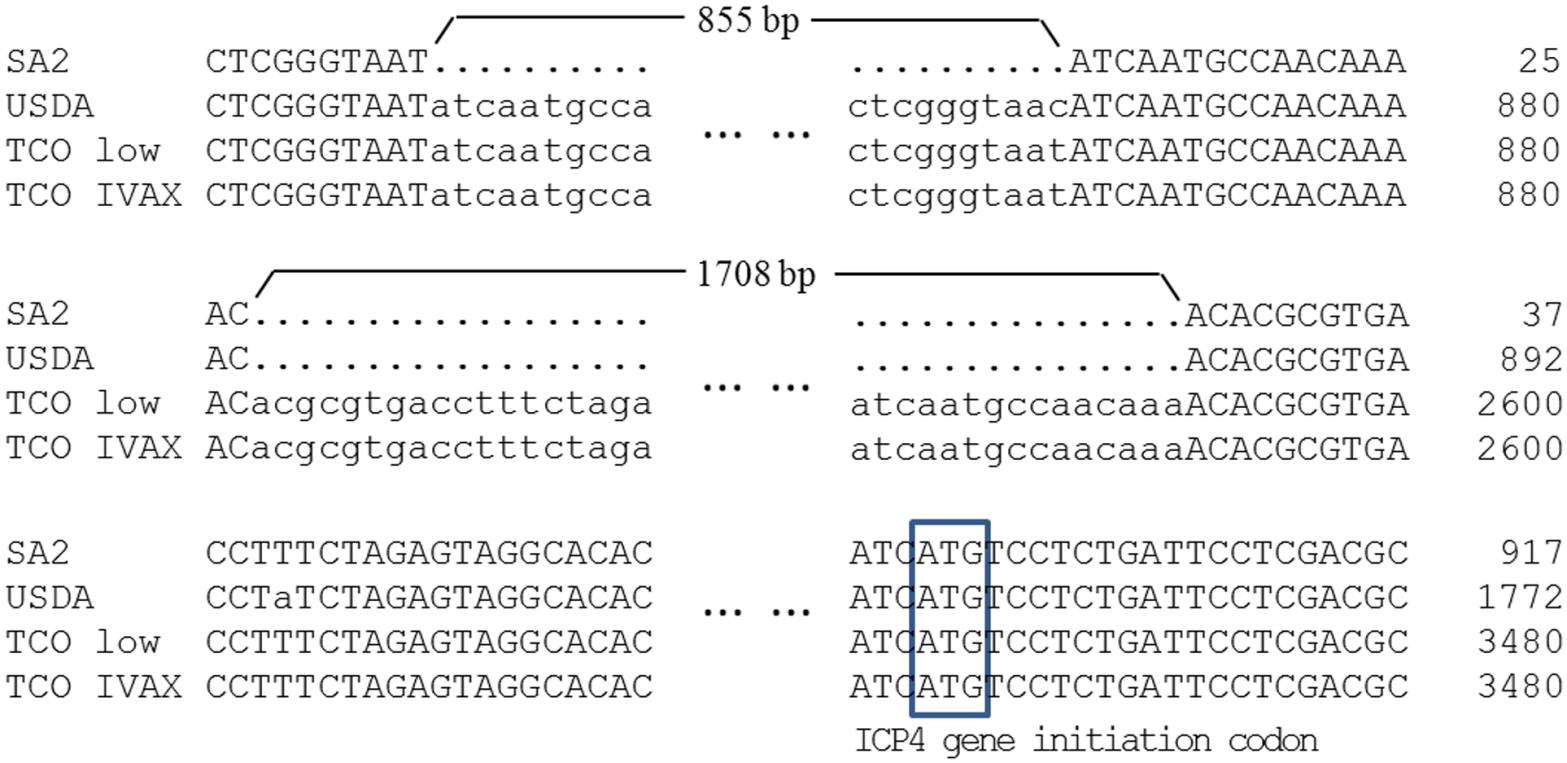
Schematic diagram of the differences in the length of the IRs regions between strains. The alignment of partial sequence in IRs region of four representative ILTV strains are shown. Compared with SA2 strain, the USDA, TCO-low, and TCO-IVAX showed an 855 bp insertion in the first part, while the two TCOs showed an additional insertion of 1708 bp in the second part. The insertion sequences were present upstream of the start codon of ICP4 gene. The other eighteen strains that are not shown in this figure were concordant to SA2 strain.

**Fig 3:**
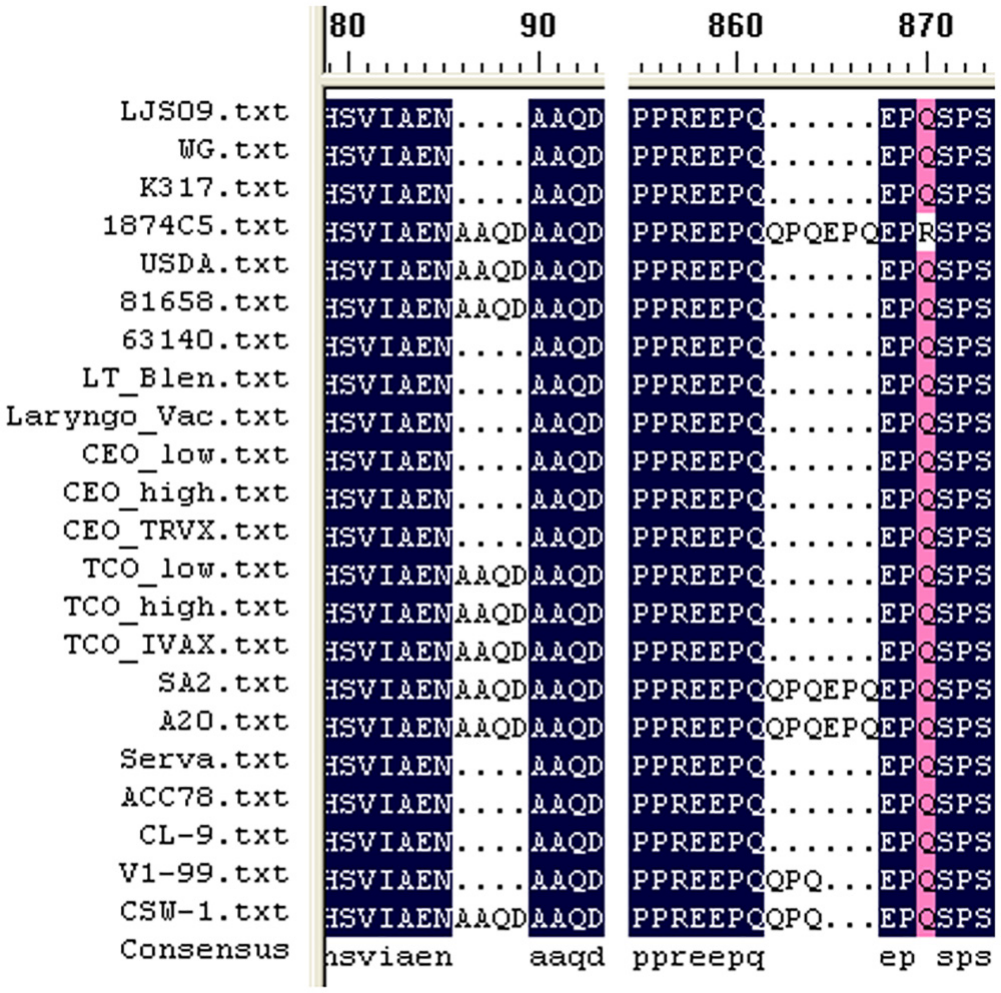
Insertions within the ICP4 amino acid sequences of some ILTV strains. The amino acid residues AAQD at 87 to 90 aa, and the residues QPQ at 862 to 864 aa, or the residues EPQ at 865 to 867 aa were found in ICP4 aa sequences of some strains.

The sequences in IRs region of the ILTV strains were different in size (Fig 1B) and showed identity of 94.2%. The IRs regions of the majority of strains were approximately 12 to 13 kb in length, this difference was mainly due to differences in the definition of the initiation sites mentioned above, and the deletion sequence in upstream or downstream non-coding region of the ICP4 gene in certain strains (WG and LJS09 strains deletion of 200bp, respectively; 1874C5 strain deletion of 1570 bp; CSW-1 strain deletion of 460 bp). However, three American strains were much longer in the IRs regions. The USDA strain was 14.5 kb whilst the TCO low passage and TCO IVAX strains were more than 16 kb due to a 855 bp and a 2563 bp insertion in the upstream of the start codon of ICP4 gene, respectively (Fig 2). The largest open reading frame in the IRs region is the ICP4 gene, which showed high similarity in amino acid (aa) sequences (up to 99.14%). Two insertions were found in ICP4 aa sequences. One is the residues AAQD at 87 to 90 aa of nine strains (1874C5, USDA, 81658, and the three TCO strains from the United States, SA2, A20, and CSW-1 from Australia), which formed double AAQD in this area with the conserved aa residues AAQD at 91 to 94 aa in all the twenty-two strains. The other insertion is the residues QPQ at 862 to 864 aa in strains 1874C5, SA2, A20, V1-99, and CSW-1 Three of them showed additional residues EPQ at 865 to 867 aa, which formed four (E/Q)PQ arrangement in this area with the double conserved residues EPQ at 859 to 861 aa and 868 to 870 aa in all the strains (Fig 3).

The Us regions of all the ILTV strains were basically consistent in size and showed identity of 99.6%. Only four strains were 30 bp longer than the others, including the Chinese strain WG, and the three Australian strains (SA2, A20, and CSW-1) (Fig 1C).

### Comparison of the amino acid sequences of glycoproteins among the twenty-two ILTV strains

The amino acid sequence of the twelve glycoproteins were aligned using DNAMAN. A total of 116 amino acid mutational sites were found in the twelve glycoproteins, the majority of which were distributed in glycoprotein B (gB), gD, gG and gJ (Table 2). Seven mutational sites were present among the Chinese, American, and Australian strains. Sixty-nine of the 116 sites existed only in strains from a single country (China, Australia, or the United States). Among which 22 sites were only found in the Chinese strains, and 34 sites were found only in the Australian strains. Another 13 mutational sites were found only among American strains, including a deletion site in the 809^th^ amino acid (Glu) of gB protein, which was absent from the American virulent field strain 61340. Furthermore, 42 sites were found in individual strains. Among which, 24 sites were found in Chinese strains: WG (9), K317 (7), and LJS09 (6); 8 sites were present in American strains: 1874C5 (5), and 61340 (3); the other 12 sites were in Australian strains CSW-1 (9), Serva (1) and V1-99 (2), respectively.

**Table 2:** Comparison of amino acid mutational sites in twelve glycoproteins of the twenty-two ILTV strains.

ORF	Amino acid sites	Conserved amino acid	LJS09	K317	WG	SA2	A20	Serva	ACC78	CL-9	V1-99	CSW-1	1874C5	USDA	81658	63140	LT Blen	Laryngo	CEO Low	CEO High	CEO TRVX	TCO Low	TCO High	TCO IVAX
gB	44	R			***H***	***H***	***H***		***H***	***H***	***H***	***H***												
gB	71	R	K	K																				
gB	116	A	V					V																
gB	207	F	L																					
gB	242	Y										**H**												
gB	348	M													$\dot{\text{T}}$							$\dot{\text{T}}$	$\dot{\text{T}}$	$\dot{\text{T}}$
gB	496	R														$\dot{\text{H}}$								
gB	551	M			***V***	***V***	***V***		***V***	***V***														
gB	644	I			T	T	T		T	T	T	T	T	T		T								
gB	707	F			S																			
gB	799	P											**$\dot{\text{S}}$**											
gB	805	K											$\dot{\text{R}}$											
gB	809	E														-								
gC	17	S									N	N												
gC	76	Y		C																				
gC	108	I										**T**												
gD	137	V				L	L				L	L												
gD	141	P				S	S				S	S												
gD	145	V									L	L												
gD	155	F	S																					
gD	158	T				N	N				N	N												
gD	194	Q				R	R																	
gD	222	D		V	V																			
gD	234	G				R	R				R	R												
gD	311	D										**N**												
gD	350	M									I	I												
gD	376	R	H																					
gE	210	R	K	K				K	K	K							K		K	K	K			
gE	267	V			***A***	***A***	***A***				***A***	***A***												
gE	386	T												$\dot{\text{A}}$	$\dot{\text{A}}$							$\dot{\text{A}}$	$\dot{\text{A}}$	$\dot{\text{A}}$
gE	420	P										**L**												
gE	424	N									**S**													
gG	45	I			*L*	*L*	*L*																	
gG	58	V			***G***	***G***	***G***				***G***	***G***												
gG	73	E										**D**												
gG	98	H			N	N	N				N	N	N											
gG	113	A			V																			
gG	115	V			G	G	G				G	G	G											
gG	118	A			*V*	*V*	*V*																	
gG	121	A									V	V												
gG	129	Q			*H*	*H*	*H*																	
gG	220	G											$\dot{\text{L}}$											
gG	262	E			G																			
gG	274	R										**K**												
gG	284	F			***L***	***L***	***L***				***L***	***L***												
gG	291	Q			*R*	*R*	*R*																	
gH	355	E			G																			
gH	401	H			***R***	***R***	***R***		***R***	***R***	***R***	***R***												
gH	452	D			G																			
gH	455	Y		**H**																				
gH	566	I			***V***	***V***	***V***			***V***														
gH	568	L			S	S	S			S	S	S	S											
gH	605	N			***H***	***H***	***H***			***H***														
gH	697	Q			R																			
gH	711	L		R																				
gH	778	R									**S**													
gI	14	T	A																					
gI	38	H										**R**												
gI	39	I			***V***	***V***	***V***				***V***	***V***												
gI	40	V										**A**												
gI	109	E	V																					
gI	178	A			D	D	D				D	D	D											
gI	185	L			***F***	***F***	***F***				***F***	***F***												
gI	211	A			*T*	*T*	*T*																	
gI	212	T			***P***	***P***	***P***				***P***	***P***												
gI	224	V			*I*	*I*	*I*																	
gI	253	P									H	H												
gI	358	D									E	E												
gJ	8	R				H	H				H	H												
gJ	74	I										T												
gJ	88	G		E																				
gJ	162	R												$\dot{\text{W}}$	$\dot{\text{W}}$							$\dot{\text{W}}$	$\dot{\text{W}}$	$\dot{\text{W}}$
gJ	163	G										**D**												
gJ	257	P									L	L												
gJ	264	M		L																				
gJ	293	V												$\dot{\text{A}}$	$\dot{\text{A}}$							$\dot{\text{A}}$	$\dot{\text{A}}$	$\dot{\text{A}}$
gJ	301	M			***I***	***I***	***I***				***I***	***I***												
gJ	318	M		K																				
gJ	337	R			*G*	*G*	*G*																	
gJ	340	A														$\dot{\text{T}}$								
gJ	359	T									P	P												
gJ	426	T			*A*	*A*	*A*																	
gJ	463	A									V	V												
gJ	494	A			***V***	***V***	***V***				***V***	***V***												
gJ	502	A			*D*	*D*	*D*																	
gJ	520	Q			P																			
gJ	584	R									W	W												
gJ	600	E			*G*	*G*	*G*																	
gJ	643	P			S																			
gJ	647	T			*I*	*I*	*I*																	
gJ	661–670	-			***STVPEITQTP***	***STVPEITQTP***	***STVPEITQTP***					***STVPEITQTP***												
gJ	694	A											$\dot{\text{T}}$											
gJ	715	V									M	M												
gJ	719	P									Q	Q												
gJ	887	L			***F***	***F***	***F***				***F***	***F***												
gK	216	V				I	I																	
gL	8	P												$\dot{\text{Q}}$	$\dot{\text{Q}}$							$\dot{\text{Q}}$	$\dot{\text{Q}}$	$\dot{\text{Q}}$
gL	126	P				Q	Q			Q														
gL	141	P						**Q**																
gL	202	A									V	V												
gM	23	A	T	T	T				T										T	T	T			
gM	32	C			Y																			
gM	229	P											$\dot{\text{S}}$											
gM	236	M		T																				
gM	350	K	E																					
gN	16	V			*M*	*M*	*M*																	
gN	40	Y			*H*	*H*	*H*																	

Note:

1. Bold (except bold italic) (**A)** represents the mutational sites were found only in single strain.

2. Italic (*A)* represents the mutational sites were found in strains WG, SA2, and A20.

3. Bold italic (***A)*** represents the mutational sites were found among WG strain and Australia strains.

4. Underline (A and **A)** represents the mutational sites were found only in Australia strains.

5. Character border (A and A) represents the mutational sites were found in Chinese strains.

6. Emphasis mark ($\dot{\text{A}}$ and $\dot{\text{A}}$) represents the mutational sites were found only in USA strains.

7. Light grey (A) represents the mutational sites were found among Chinese strains, USA strains and Australia strains.

It is noteworthy that a total of 13 mutational sites were found only among strains WG, SA2 and A20, and these 13 mutational sites were mainly concentrated in four glycoproteins including gG, gI, gJ, and gN. An additional 15 sites were found among the Chinese strain WG and some Australian strains, including a 10 aa insertion in the 661^th^-670^th^ of gJ among strains WG, SA2, A20, and CSW-1.

### Analyses of the OriS regions of the twenty-two ILTV strains

**Fig 4:**
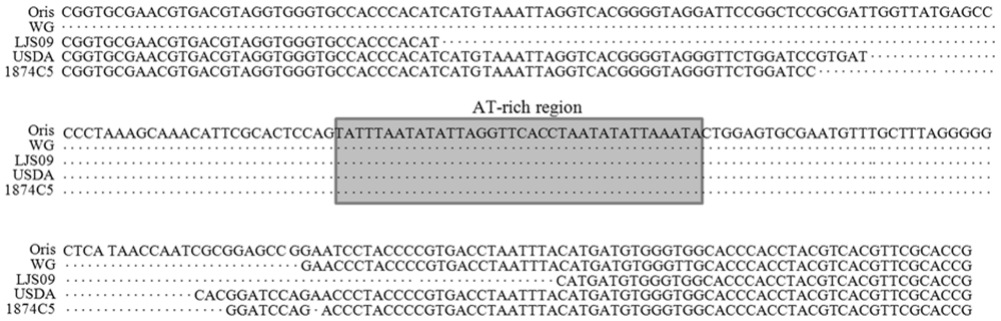
Deletions of the OriS region in four ILTV strains. The complete OriS sequence was 266 bp [GenBank: AM238250]. When compared with the complete OriS sequence, four of the twenty-two strains showed deletions, including strains WG (65 bp), LJS09 (76 bp), USDA (152 bp), and 1874C5 (144 bp). All these four strains did not contain the AT-rich region. The other eighteen strains had complete OriS sequences.

Analyses of the OriS region of the twenty-two ILTV strains with the OriS sequence [GenBank: AM238250] showed that eighteen ILTV strains contained complete OriS sequence (266 bp), while only four strains showed deletions, including strains WG (65 bp), LJS09 (76 bp), 1874C5 (144 bp) and USDA (152 bp). All these four strains did not contain the AT-rich region (Fig 4).

### Phylogenetic analysis of nucleotide sequence in complete genome and different subregions of the twenty-two ILTV strains

**Fig 5:**
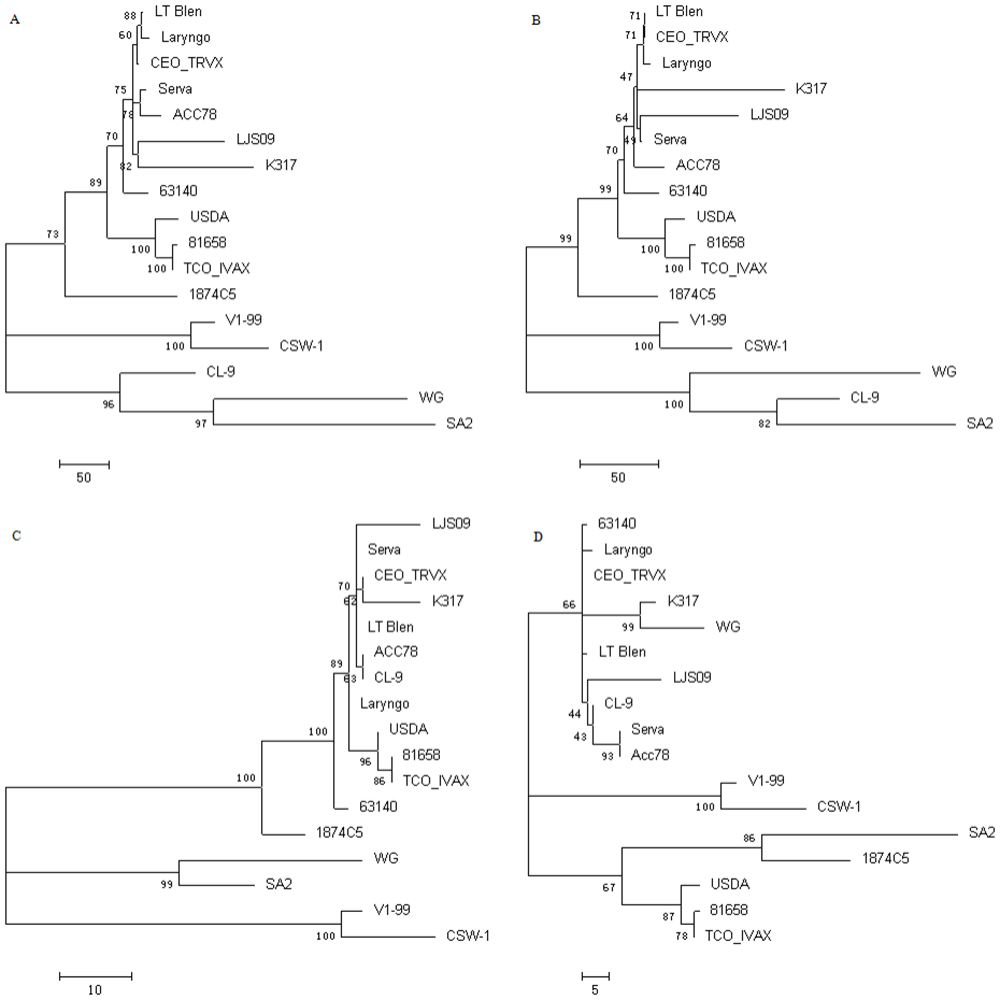
Phylogenetic tree analyses in the complete genome and different subregions of representative ILTV strains. The phylogenetic trees generated from the alignments on the whole genome (A), the U_L_ region (B), the Us region (C), and the IRs region (D) of seventeen of the twenty-two ILTV strains using Maximum Parsimony method in MEGA program. One hundred bootstrap replications were used to assess the significance of the tree topology. A bar indicates the sequence substitutions per site. To avoid incorrect divergence information, five artificially passaged strains were not included in the analysis (CEO low, CEO high, TCO low, TCO high, and A20).

To better understand the relationships among the individual ILTV strains isolated from different periods of time and different regions (China, USA, and Australia), alignments on complete genome sequences and the sequences in different subregions (U_L_, Us, and IRs) were performed using MAFFT. The phylogenetic trees were analyzed using 17 out of the 22 ILTV strains. To avoid incorrect divergence information, five artificially passaged strains were not included in the analysis, which were strains CEO low and CEO high, TCO low and TCO high, and A20. Those strains were the different passages of CEO TRVX, TCO IVAX, and SA2, respectively. The establishment of phylogenetic trees based on the whole genome and the genomic sequences in different subregions formed three major clusters, respectively. In the tree generated from the whole genome, the Chinese strain WG and the Australian strains SA2 and CL-9 were divided into one cluster; V1-99 and CSW-1 strains from Australia formed a single cluster; and the remaining twelve strains formed a cluster, composed by three branches. One main branch includes the two Chinese strains (K317 and LJS09), three American vaccine strains (CEO TRVX, LT Blen, and Laryngo), the European origin vaccine strain (Serva), and two virulent field strains from Australia (ACC78) and the United States (63140), respectively. Another branch contains three strains from the United States, including two virulent field strains (USDA and 81658) and the TCO IVAX vaccine strain. The American strain 1874C5 formed a single branch in this cluster (Fig 5A). The phylogenetic trees established according to the sequences in the different subregions (U_L_, Fig 5B; Us, Fig 5C; and IRs, Fig 5D) were generally similar to that of the complete genomes.

By phylogenetic analysis, the evolutionary relationship between the two Chinese strains K317 and WG were determined. K317 was always close to the American vaccine strain CEO and its derivatives. Interestingly, WG strain showed very close relationship to the Australia vaccine strain SA2 in the whole genome, U_L_ region, and Us region. Overall, there were no obvious regional divergences in the evolutionary relationships among the twenty-two strains from China, Australia and the United States.

### Possibility of recombination in complete genome between ILTV strains

**Fig 6:**
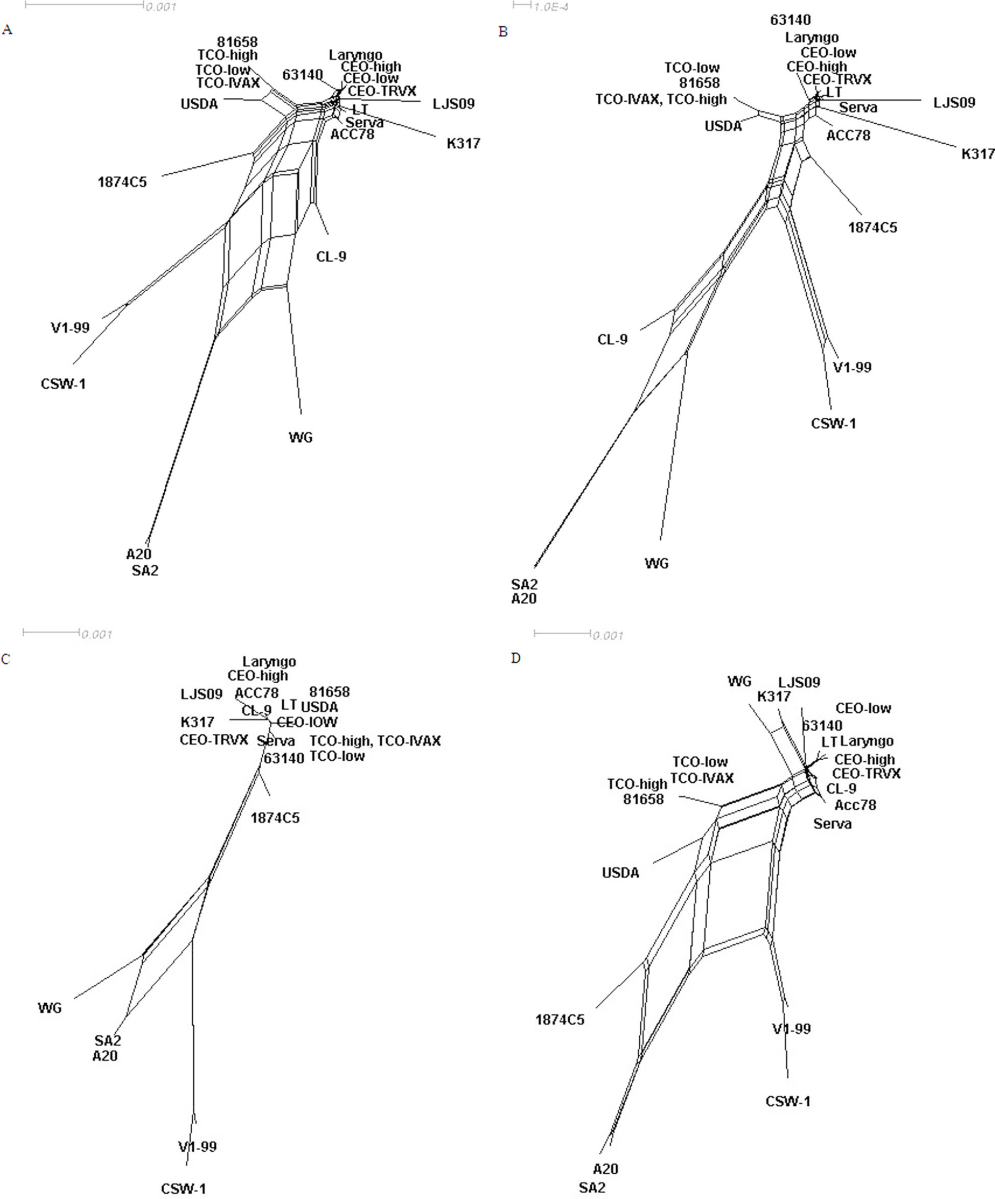
The recombination networks among all the ILTV strains. Phylogenetic networks from nucleotide sequence alignments on complete genomes (A), the U_L_ region (B), the Us region (C), and the IRs region (D) of the twenty-two ILTV strains were generated using SplitsTree. The multiple reticulate networks indicate the recombination events among ILTV strains. A bar indicates the sequence substitutions per site. The phi test did find statistically significant evidence for recombination (p < 0.001) in complete genome, the U_L_ region, and the IRs region, whereas the evidence for recombination in the Us region was not significant (p > 0.05).

The phylogenetic analysis mentioned above did not group strains isolated from similar geographical regions together. This may be due to the high similarity of genomic sequences between isolates, or the possibility of a natural recombination before they were isolated, as has determined among Australian strains[9]. Recombination networks of the whole genome and different subregions of the twenty-two ILTV strains were performed (Fig 6). As expected, a total of 971 informative sites were found in the whole genome of the twenty-two ILTV strains, and the phi test did find statistically significant evidence for recombination (p < 0.001) (Fig 6A). The recombination events were also detected in U_L_ region (518 informative sites, p < 0.001) (Fig 6B) and IRs region (157 informative sites, p < 0.001) (Fig 6D). However, 131 informative sites were found in Us region, but the evidence for recombination was not significant (p = 0.056) (Fig 6C). The topologies of phylogenetic networks in complete genome and in different subregions of all the ILTV strains generated by SplitsTree were consistent with the relationships analyzed by MEGA.

**Fig 7:**
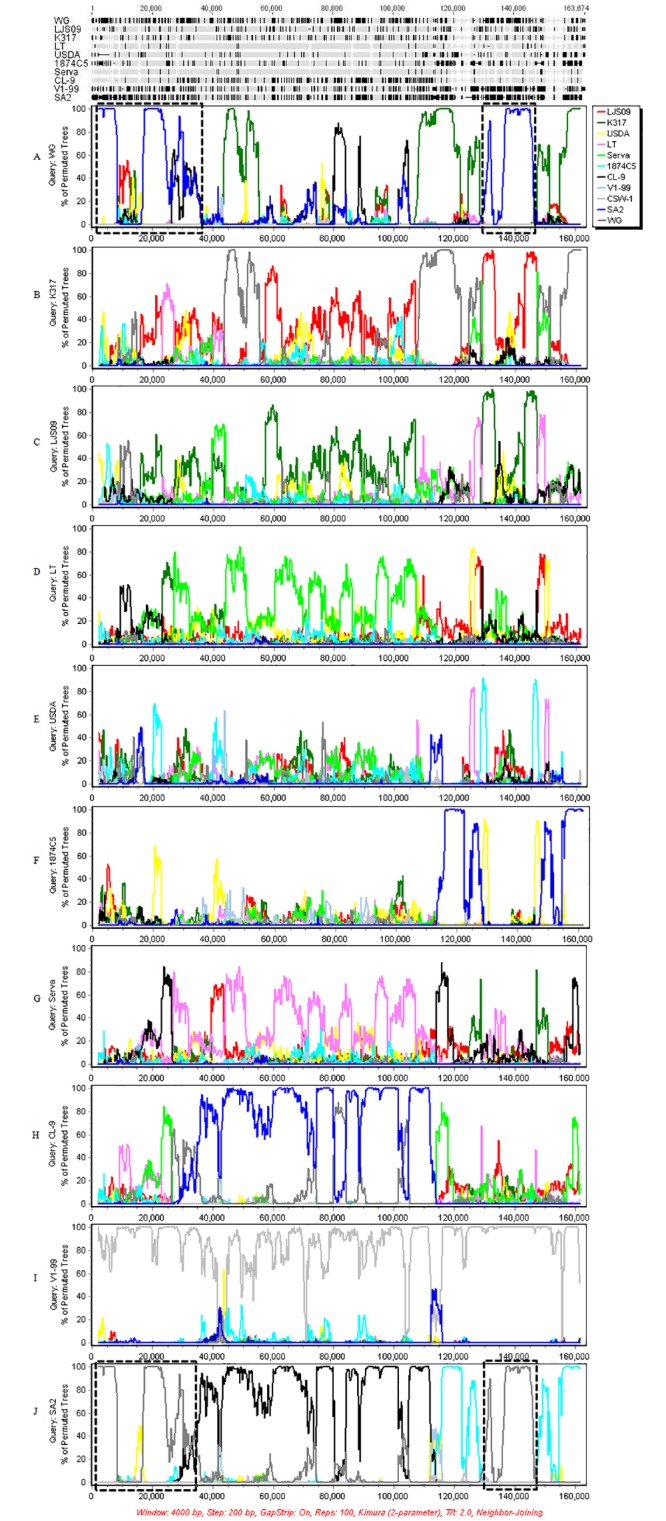
Bootscan analyses of recombination crossover points in representative ILTV strains. Aligements and Bootscan analysis were performed using Simplot program with ten complete genome sequences as query sequences WG (A), K317 (B), LJS09 (C), LT (D), USDA (E), 1874C5 (F), Serva (G), CL-9 (H), V1-99 (I), and SA2 (J), which were selected as representative strains in each branch of the recombination networks. The crossover points were detected in most strains as query sequence. The regions near the 5 'end and around the Us region of WG and SA2 strains are marked in a black box.

**Fig 8:**
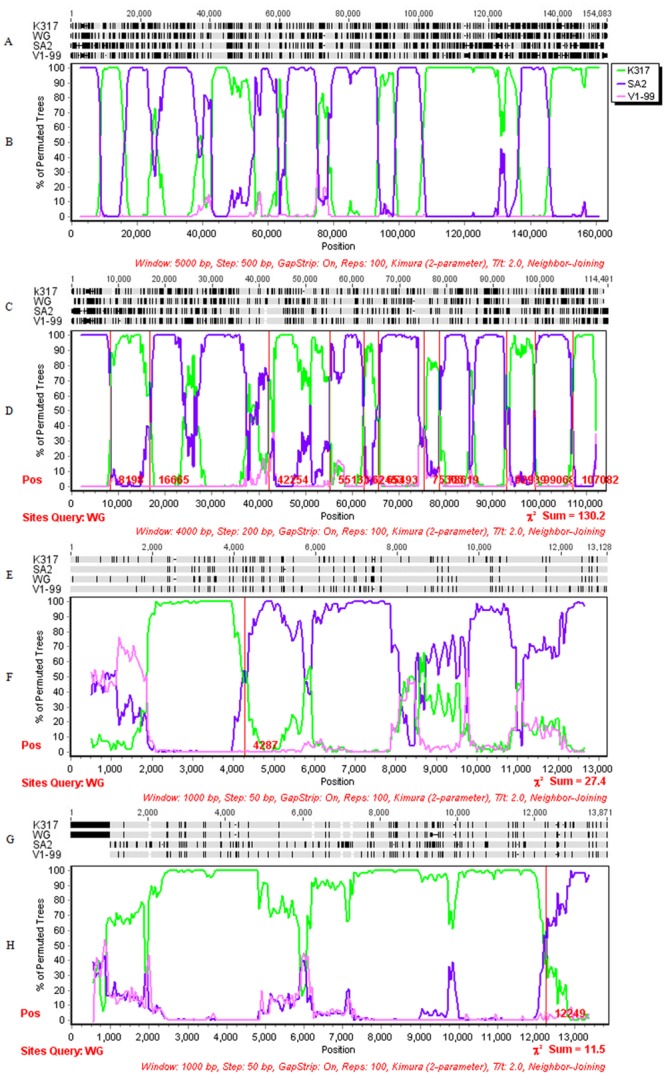
Bootscan analysis of crossover points in different subregions of WG strain with reference strains. Alignments and Bootscan analyses were produced in complete genome (A, B), the U_L_ region (C, D), the Us region (E, F), and IRs region (G, H) using WG strain as query sequence, SA2, K317 and V1-99 strains as parental sequences or control, respectively. The crossover points of predicting recombination events are marked on the Bootscan maps of different subregions. The Chi-square (x^2^) value of heterogeneity between adjacent regions is shown on the bottom right of each map (p < 0.001). All values have 1 degree of freedom.

To further determine the recombination events among the available individual ILTV strains, Bootscan analysis of several strains (WG, K317, LJS09, LT, USDA, 1874C5, Serva, CL-9, V1-99 and SA2) selected as a representative for each branch was used to search for the crossover points for the whole genomes with 4000 bp window size and 200 bp step size. The crossover points were detected in most strains as query sequence (Fig 7). When WG strain was selected as query, two potential parental strains (K317 and SA2) were prominent in the map (Fig 7A). Further Bootscan analysis was performed using WG strain as query sequence, K317 and SA2 strains as potential parental sequences, and V1-99 strain as a control. The map showed a clear picture that WG strain was generated from the two selected parental sequences K317 and SA2 in multiple crossover points throughout the whole genome (Fig 8A and 8B), and the proportion of SA2 strain was relatively larger than that of K317 strain.

### The evidence of recombination event in different subregions of WG strain

To further determine the recombination events in different subregions (U_L_, Us, and IRs) of WG strain. Bootscan analysis were performed using the four selected strains (WG, K317, SA2, and V1-99) with the crossover points of putative recombination events mapped by examining the distribution of phylogenetically informative sites supporting alternative tree topologies.

In the U_L_ region, multiple breakpoints were found (Fig 8C and 8D). A total of 197 informative sites in alignment with the supported phylogenetical trees were found in this region. The Chi-square (x^2^) test for heterogeneity between adjacent regions of the distribution of informative sites supporting different trees (p < 0.001) are showed at the bottom right of the figure. All values had 1 degree of freedom. The x^2^ Sum value was up to 130.2.

A predicting recombination site located nearby 12249 was found in the IRs region (x^2^ Sum = 11.5, p < 0.001) (Fig 8H). It should be noted that the four sequences involved in the analysis were different in length (Table 1) so the position of predicting recombination sites may have deviation. Due to the difference in starting position of the four strains in IRs region, the 5 'end of WG and K317 strains were 1022 bp longer than that of SA2 strain. Alignment of the three strains in this subregion suggested that WG strain and K317 strain showed high similarity from 1–12255 bp. Except for the conserved sequences among the three strains, 1785 common sites were found, including the 1022 bp in the 5' end, and the remaining 763 sites were found, including 11 insertions and 39 deletions. From 12321 to 3' end, WG strain showed high similarity with SA2 strain, with 31 common sites, including 11 insertions and 8 deletions. (Fig 8G). The junction of the similarity sequence (12255–12321) was defined as recombination sites, and were consistent with the results in Bootscan analysis. Twenty-four informative sites with the supported phylogenetical trees were found in this region.

**Fig 9:**
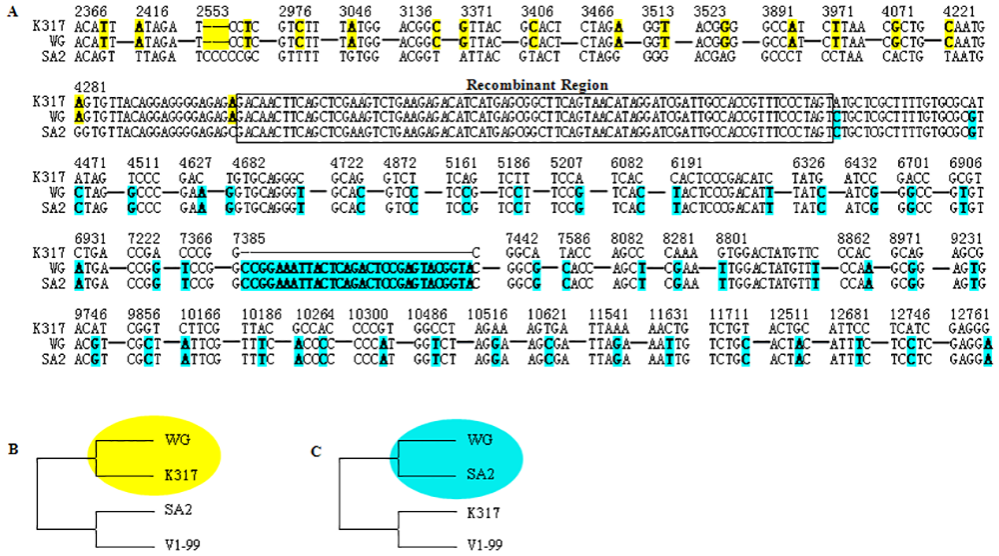
Alignment analysis of breakpoint in Us region of WG strain. The breakpoints analysis were performed by using WG strain and the two potential parental strains SA2 and K317 (A). The unique sites between WG and K317 strains are marked in yellow background, while the unique sites between WG and SA2 strains are marked in blue background. The breakpoints are marked in a black box. The phylogenetic tree generated by the sequences nearby the unique sites shows close relationships with K317 strain (B) and SA2 strain (C), respectively.

Surprisingly, the sequences close to 4287 bp in Us region of WG strain was found to be a predicting crossover point (x^2^ Sum = 27.4, p < 0.001) (Fig 8E and 8F). Even though, there was no significant evidence for recombination in Us region of all the strains (p = 0.056) (Fig 6C). A total of 37 informative sites with the supported phylogenetical trees were found in this region. To further confirm the recombination event of WG strain, the breakpoints analysis were performed by using WG strain and the two potential parental sequences. The sequence comparison of strains WG, K317 and SA2 showed high similarity up to 99.64%. From 2300 bp to 4301 bp, the WG strain showed 20 unique sites to K317 strain; while from 4382 bp to the 3' end, the WG strain showed 78 unique sites to SA2 strain. Therefore, the similar junction (4302–4381) was defined as breakpoint (Fig 9A). The phylogenetical trees performed by the sequences close to the 20 sites and the 78 sites were entirely consistent with the results of informative sites analysis (Fig 9B and 9C).

## Discussion

In this study, the genomes of two Chinese strains WG and K317 were sequenced using sanger sequencing techniques, all the fragments were amplified by conventional PCR method and modified SON-PCR method as described previously [35]. The two genomes of WG and K317 strains were assembled to be 153505 bp and 153639 bp in length, respectively. The two strains were basically consistent with other twenty published genome sequences of ILTV in size (Table 1).

Virus glycoproteins play an important role in virus adsorption and penetration of the host cell, virus transmission between cells, mediating immune response and inducing neutralizing antibodies [48–52]. A total of 116 amino acid mutational sites were found in the twelve glycoproteins among all the ILTV strains. The characteristic mutations among the Chinese strain WG and the Australian strains were approximately up to a quarter of the total mutational sites (Table 2). Especially between WG strain and strains of SA2 and A20. Beside the mutation sites in glycoprotein sequences, an additional thirty-eight mutational sites were found only among the above three strains distributed in eighteen genes in the whole genome, including open reading ORF C, ORF D, ORF E, sORF4/3, Tk, U_L_5, U_L_7, U_L_15, U_L_17, U_L_20, U_L_36, U_L_38, U_L_39, U_L_46, U_L_48, Us3, Us8A, and Us10, respectively (S1 Table).

ILTVs have three origins of viral DNA replication, two copies of OriS located in the two repeated regions and one copy of OriL located in the U_L_ region [33]. In this study, comparison analysis of all the twenty-two ILTV strains showed intact OriL sequences and eighteen strains possessed intact OriS sequences, while only four strains had deletions in the OriS sequence, in which the shortest OriS sequence was from WG strain (65/266 bp) (Fig 4). Although we did not compare the impact of deletions in OriS sequence on replication kinetics, the results from other researchers with HSV-1 showed that the deletion of both copies of OriS sequence may have little effect on viral replication *in vitro* [53, 54].

Some researchers have found that it is crucial to study the epidemiology of ILTV as CEO-vaccine-related ILTV isolates have frequently been identified as a cause of disease [5, 55–57]. The comparison of the ICP4 gene in IRs region of all the ILTV strains found two insertion sites. One is the residues AAQD at 87 to 90 aa, which were found in TCO strains and some virulent field strains from the United States and Australia, but was absent in the CEO strains and its related isolates. The other insertion residues QPQ(EPQ) did not exist in the CEO strains, either. The insertion of amino acid residues in ICP4 gene could be used as a marker to distinguish the TCO strains from the CEO strains, instead of the PCR-RFLP method [5].

In this study, the phylogenetic analysis of individual complete genome sequences of ILTV strains from China, the United States, and Australia were performed. Although there were not able to find obvious regional divergences in the evolutionary relationship among the strains from different geographical regions, this study showed close relationships between the Chinese strain WG and the Australian strains SA2 in many aspects, including more than 50 common unique amino acid sites between the two strains throughout the whole genome, and the close relationships in phylogenetic analysis. Previous studies showed that the ILTV strains isolated from backyard flocks in the USA and the Australian origin SA2 strain shared a common ancestor, and that this ancestor may have originated on the American continent and then been subsequently introduced into Australia, owing to the comparative analyses of the UL27 and ICP4 gene were similar in sequences [36]. Obtaining the complete genome sequence of the Chinese strain WG will help facilitate finding the origin of SA2 strain.

The vaccine strain SA2 has been used only in Australia [36] since SA2 was not exported to other countries. It is therefore difficult to explain the SA2-like virulent field WG strain isolated from WangGang of China in the 1950s. There is the possibility that recombination events occurred before the isolation of these two strains, or that they originated from a common ancestor, or that the WG strain is the parental strain of SA2 strain. Further analysis on possible recombination networks were performed using all the available individual ILTV strains published in GenBank. The evidence for recombination networks in the whole genome, the U_L_ region, and the IRs region of the twenty-two strains were found to be statistically significant (Fig 6A, 6B and 6D) and although the evidence for recombination in Us region of all the strains were not significant,.the recombination networks did exist between WG and SA2 strains (Fig 6C), which suggested that the possibility of recombination may exist between them. However, no recombination network was found among other reference strains, resulting in the evidence of recombination in Us region being not significant.

Bootscan analysis of recombination event in whole genomes were performed using WG, K317, LJS09, LT, USDA, 1874C5, Serva, CL-9, V1-99 and SA2 strains as representative strains. It is surprisingly to find that the sequences near the 5 'end and around the Us region of WG and SA2 strains interchangeably appeared in the corresponding position of each other, even the shape of curve in the two region of the two strains were exactly the same (Fig 7A and 7J). It can be considered that WG strain and SA2 strain acted as parental strain of each other in certain regions of the whole genome. The K317 strain was another strain appearing in multiple regions of the map that using WG strain as query strain. At least four pairs of recombination crossover points in whole genome were detected by Bootscan analysis using WG strain as query, SA2 and K317 strains as parental strains, and V1-99 as control. Further analysis in different subregions of WG strain revealed that crossover points can be found in all the three subregions. It is worth mentioning that the Bootscan analysis further supported our previous assumption, confirming that recombination event did exist in the Us region of certain strains (Fig 8F). In this study, the evidence of recombination event in Chinese virulent field strain WG was detected by Bootscan analysis with the sequences of two vaccine strains K317 and SA2, which supported the viewpoint of previous studies that found attenuated vaccines can recombine to form virulent field viruses [9], and evidence of historical recombination events between two distinct viral lineages [36]. The vaccine strains K317 has been used in different countries, however the SA2 vaccine strain has been used only in Australia, so the parent strain of the WG strain might be derived from the SA2-like strain.

## Conclusions

In this study, two new complete genome sequences from China were determined by Sanger sequencing method. The two new complete genome sequences of Chinese ILTV strains were used in comparative analysis with other individual complete genome sequences of ILTV strains from China, the United States and Australia. The analysis of the genome sequences, phylogenetic trees, and recombination events showed close relationships between the Chinese strain WG and the Australian strain SA2. More information about individual complete genome sequences from different geographic regions will help to facilitate the analysis of phylogenetic relationship and the molecular differences among ILTV strains.

## Supporting Information

S1 Table Mutational sites among strains WG, SA2, and A20 distributed in the whole genome beside the glycoproteins. (DOCX)
