# Supplementary material for: Multiple Comparison Analysis of Two New Genomic Sequences of ILTV Strains from China with Other Strains from Different Geographic Regions
Source: PLoS One. 2015 Jul 17;10(7):e0132747. doi: 10.1371/journal.pone.0132747 (PMC4505947; doi:10.1371/journal.pone.0132747)
Supplement: S1 Table — (DOCX) [file pone.0132747.s001.docx]

Table S1 Mutational sites among strains WG, SA2, and A20 distributed in the whole genome beside the glycoproteins

| ORF | Amino acid sites | Conserved amino acid | SA2 | A20 | WG |
| --- | --- | --- | --- | --- | --- |
| ORFC | 67 | - | D | D | D |
| ORFC | 89 | H | R | R | R |
| ORFC | 181 | L | P | P | P |
| ORFD | 16 | P | L | L | L |
| ORFD | 272 | V | D | D | D |
| ORFE | 102 | P | A | A | A |
| ORFE | 132 | I | L | L | L |
| ORFE | 227 | I | T | T | T |
| ORFE | 245 | Q | R | R | R |
| sORF4/3 | 277 | I | M | M | M |
| TK | 252 | T | M | M | M |
| UL5 | 572 | G | E | E | E |
| UL5 | 738 | K | R | R | R |
| UL7 | 344 | R | H | H | H |
| UL15 | 135 | A | T | T | T |
| UL15 | 220 | E | D | D | D |
| UL17 | 205 | N | T | T | T |
| UL17 | 440 | V | A | A | A |
| UL20 | 176 | K | N | N | N |
| UL36 | 1043 | R | K | K | K |
| UL36 | 1143 | S | G | G | G |
| UL36 | 1165 | T | A | A | A |
| UL38 | 249 | L | F | F | F |
| UL38 | 399 | G | R | R | R |
| UL39 | 37 | R | K | K | K |
| UL39 | 44 | A | D | D | D |
| UL46 | 209 | C | R | R | R |
| UL46 | 550 | A | T | T | T |
| UL48 | 20 | E | D | D | D |
| UL48 | 137 | N | D | D | D |
| UL48 | 176 | A | V | V | V |
| US3 | 92 | Y | S | S | S |
| US3 | 97 | P | A | A | A |
| US3 | 119 | G | D | D | D |
| US3 | 172 | N | S | S | S |
| US8A | 88 | S | F | F | F |
| US8A | 154 | G | D | D | D |
| US10 | 227 | C | R | R | R |
